# Older People Negotiating Independence and Safety in Everyday Life Using Technology: Qualitative Study

**DOI:** 10.2196/10054

**Published:** 2018-10-19

**Authors:** Randi Stokke

**Affiliations:** 1 Department of Health Sciences Norwegian University of Science and Technology Gjøvik Norway

**Keywords:** welfare technology, patient care, the social alarm, caring practices, home care services, science and technology studies, public service innovation, co-production

## Abstract

**Background:**

Due to demographic changes with an aging population, there is a demand for technology innovations in care services. However, technology innovations have proven difficult to implement in regular use. To understand the complexity of technology innovations in care practices, we need a knowledge base of the complex and diverse experiences of people interacting with established technologies.

**Objective:**

This paper addresses the research gap in relation to understanding the microcontext of co-production of care involving established technologies integrated into care practices. The paper also aims to provide a framework for exploring what really happens when different actors use technology in care practices.

**Methods:**

Participant observations and 22 interviews with actors using social alarms were conducted employing the critical incident technique. A stepwise deductive-inductive analysis was then performed.

**Results:**

The results reveal how co-production of care assumes different meanings according to how actors use the technology. The results also show how technology innovation changes the dynamics between the actors and rearranges care practices. Independent and safe living is co-produced through performing bricolages and optimizing practice. Additionally, this opens up for unexpected results and bricolages as an integrated part of technology innovations.

**Conclusions:**

This study illustrates how care services are always co-produced between the actors involved. By using aspects from science and technology studies, this paper provides a framework for exploring technology in use in care practices. The framework provides tools to unpack and articulate the process of co-producing services.

## Introduction

### Technology Innovations in Caring Practices

There is a persistent demand in public policy in western societies for increased technology innovation in community health care to meet challenges in the services caused by the “silver tsunami” and to facilitate active aging and independent living [1,2].

Technologies intended for care practices are often advocated as plug and play solutions to the challenges of facilitating safety and independent living and to avoid or postpone nursing home admission [3]. However, there is a discrepancy between these expectations and the complex reality in which these technologies are embedded [4-6]. The integration of technologies has proven difficult, and experiences so far have shown that it is very difficult to progress from pilot projects to regular use and then to scale up to other contexts [4,7-9].

There are many studies describing the effects of technology innovations in care practices [10] that attempt to identify drivers and barriers. However, such research seldom captures what happens after the pilot phase [11]. As the knowledge base is rather sparse, we need a more comprehensive understanding of how technologies integrated into health care practice work [10]. Moreover, we need a greater understanding of the practice of using established and integrated technology in regular service. My paper explores this by studying the use of the social alarm, an established technology that aims to provide safety and independent living for older people living at home. Focusing on this well-integrated and widely used technology allows us to study the emergence of personal, professional, and organizational issues that are little seen in new technology innovations. This is done by examining how people involved in using this technology co-produce safety and independent living within care practices and how they interact with the technology involved.

To address the research gap in understanding the complex work of established technologies in care practices, this paper positions itself within the newer public service innovation tradition that offers ways of understanding how public services are always co-produced by the actors involved in the service organization as an inevitable part of the service [12,13]. There has been little focus on the different actors’ role, interaction, and co-production in a microcontext within the service innovation literature [13], and this paper contributes theoretically by suggesting a framework for exploring co-production in a microcontext, using constructs from science and technology studies (STS). In addition, it provides tools to unpack the process of co-production and address how this contributes to safety and independent living for older people with the social alarm.

### The Social Alarm

Despite the major focus on technology innovations in care practices, social alarms still form the bulk of technologies in use in care practices and are widely in use in western societies [4,14]. The aim of a social alarm is to contribute to safety and independent living for frail older people. Even though well established and used since the late 70s, a previous review of social alarms illuminates diverse experiences and issues related to their use [15].

**Figure 1:**
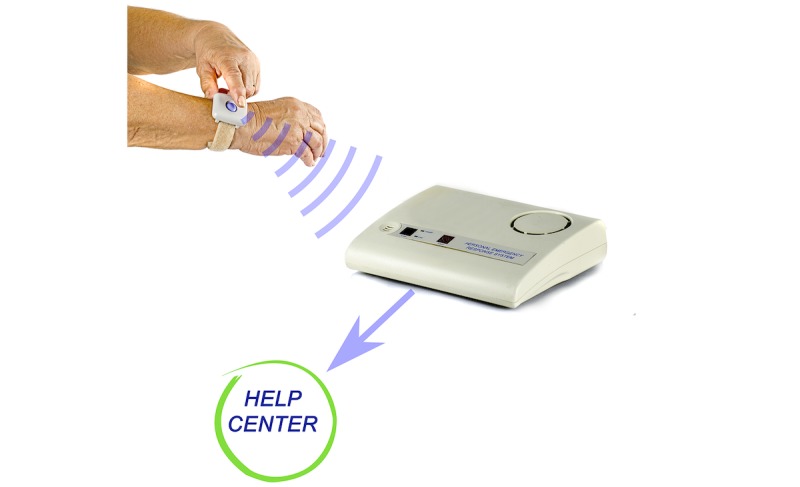
Illustration of the social alarm.

A social alarm consists of a unit placed centrally in the home and a pendant or wrist-worn device the end user can press when in need, as shown in Figure 1, to summon help from a dedicated responder.

Many actors are involved in the use of the alarm. The typical end user is an older dependent person living alone. Relatives, neighbors, care workers, and technology facilitators are all actors with different expectations, experiences, roles, and relationships with each other and the alarm [15,16].

Homecare workers usually offer the service needed when the social alarm is activated. As it is impossible to predict when it will be activated, the care worker might be anywhere in the homecare district at the time. The response time, therefore, varies from immediate response to several hours, depending on the number of care workers, their geographical location, and whether they are assisting other patients at the time.

The daily care practice is hard to plan, as care is unpredictable and emergent activities within a network of actors forming complex interactions. This demands flexibility and the ability to adjust plans, prioritizing some work and deprioritizing other work [17].

### Theoretical Framework

This paper will draw on aspects of service innovation studies and STS to provide a fruitful contribution by exploring how the different actors involved co-produce safety and independent living for the end user of the social alarm. Innovation studies, including service innovation studies, and STS are two major adjacent fields inspired by many of the same theories. Both are concerned with technology innovations, social context, and the use of knowledge. Although thematically related, they are distinct research areas with limited interaction between them [18-21]. I will use the notion of script, domestication, and heuristics from STS as tools for framing, exploring, and understanding the co-production between actors involved. I will thereby add more pieces to the puzzle of understanding the complexity of technology innovation by articulating a framework for exploring the co-production between the actors and by grounding this framework empirically.

The concept of co-production is used in different ways, and its many definitions have been criticized for being rather blurred and lacking an empirical evidence base [22]. Co-production describes the relationships between different actors, and public service innovation literature states that services are always co-produced [23]. A service organization can suggest a service, but it is in the interaction between the service provider and the service user that the actual service is co-produced. Using the concept of co-production changes the traditional way of understanding a service as provided by the care service and received by the users of the service.

People are relational entities and co-produce care through “a relational, situated and embodied achievement in which people explore the right thing to do for themselves and their relationships” [24].

A co-production of practice (eg, a practice of using a technology) can provide different value for the different actors involved, such as safety and independence for the end user. However, the co-production might also lead to destruction of value [25,26] and in the worst case, failure of the technology integration.

Care research has shown that technology in use is always interwoven in complex networks of care practices that contribute to changing, shaping, and revealing new meanings of care in unforeseen ways [3-5,10,27,28].

Accommodating an integration of technology and co-producing care for the users implies a change in the care practices. This involves new interactions and changing roles and practices, redefining how actors live, work, and even identify their lives [10,28]. Responsibility is delegated to care workers and users [29,30], redefining their role from passive recipients to active participants and requiring them to become competent users of the technology [28]. This co-production involving changes in roles and responsibilities has been largely disregarded in previous research in the field [15] and will be addressed in this paper.

The co-production is a way of empowering as well as exploiting actors [31]. The experience of value is highly contextual and directly influenced by the expectations toward a service [25]. What goes on in the process of co-production is a complex and indistinct process that we need theoretical tools to explore.

Since a user’s perception is affected by both expectations and experience with the service, the moment these two collide is the moment of truth. This moment is, in fact, a continuum, a process from expectations to experience [12,25].

The notion of script and domestication offers us tools for exploring and analyzing the practical work of co-production of care and underlines how using a social alarm can lead to values such as safety and independence for the end user.

The script metaphor describes technologists’ vision of a technology’s function, the expectations of how the users will relate to the technology and vice versa [32-35]*.* Social alarms can be scripted in different dimensions: as technological devices, as integrated services, and how values come into play for the actors involved [36].

The domestication metaphor, inspired by the process of domestication of wild animals, provides a way of exploring this continuum in depth and showing the relational process of how an object gradually becomes a part of everyday living [37,38]. This involves back and forth battles of values, pride, resistance, refusal, and tension in the interaction between the technology and humans involved [33,38]*.*

From the domestication process, a more or less stable relationship is established between the actors and the technology. Pols [10] describes this as taming and unleashing in care practices where 4 heuristics emerge. Following these relationships enables us to explore the interactions and co-production in depth.

There are examples where human-technology interaction works exactly as planned/scripted. Furthermore, sometimes the actors reject the technology altogether—there is no domestication, which causes the failure of technology projects. However, this paper focuses on technology in use. Technology in use in this context means that the technology is integrated and adopted into a care practice.

As technology innovations become an established part of practice, the focus fades and the integration process becomes more tacit [39]. You might say that the technology tends to move off the radar as it becomes domesticated. However, even if domesticated, a technology is never completely fixed. The technology as an artifact opens up for different interpretations of how to use it, what to think of it, and what feelings it inspires, etc. [40,41].

The practice of descripting and domestication described above often involve small ad hoc innovations called bricolages. These are simple, unplanned solutions using resources at hand as an answer to a problem [4,42]. Table 1, an expanded version of Pols [10], illustrates different heuristics that play out in practice involving technology in use and how this might lead to bricolage. Thus, Table 1 provides an illustration of how both the technology and human actors are active parts of the co-production of care practices involving technology.

**Table 1:** Describing heuristics of taming and unleashing technology and actors. An expanded version of Pols (2017).

Heuristics	Description of the heuristics
The techology is used in accordance with the scripts	Actors adopt, integrate, and domesticate the technology in accordance with expectations, and the technology is part of co-producing care practices.
Taming the users	Technologies sometimes tame users by making them dependent on the technology and making them adjust their lifes according to the technology.
Unleashing the users	Technologies can unleash users, making them request new services from the technology. Here the script and intention of the technology are not meeting the demands and expectations from the actors involved, thus leading to bricolage or dissatisfaction with the technology.
Taming the technology and unleashing practices	Actors tame the technologies by using them to pursue their goals, either by exploiting only some possibilities the technology offers or by finding new ways of use, often through bricolages, often in other ways than scripted and intended by designers and vendors. Sometimes the technologies unleash unexpected and completely new areas of use.
Nonuse	The users reject the technology altogether; domestication does not occur.

Acts of bricolage can add up to significant changes in routines or use of technology but they are often hidden and unarticulated in the co-production of daily practices [43]. Nurses often perform bricolage in care work to solve problems on the spot [44].

According to Star and Strauss [45] and Allen [17], care work largely incorporates this way of working where actors need to adjust the practice or the technology [4,17]. Bricolage often allows users and family members to take the initiative in co-producing care by finding better solutions [46]. By collecting bricolages and bringing them to the attention of colleagues and management, it is possible to turn them into useful innovations to improve the quality of the service [42].

### Framing Co-Production of Technology in Use

**Figure 2:**
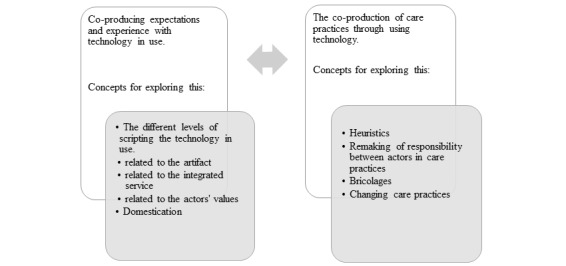
Integrated framework.

Bridging the different constructs described in an integrated framework contributes theoretically by offering ways of exploring how the co-production practice in care work involving technology might be explored, as presented in Figure 2.

This constitutes a continuum of tools for exploring and understanding the complex reality of co-produced care practices with technology in use. The model’s linearity is for analytical purposes, as the lived reality is dynamic, with processes going back and forth.

This framework provides tools for understanding the use of established technologies in care practices by adding STS concepts to explore the co-production that always takes place between the different actors involved. Later in the paper, this will be related to the data from the empirical material related to the social alarm.

The aim of the paper was to explore and interpret different actors’ experience with an established technology innovation in care practices and discover how actors use and interact with social alarms and what strategies they apply when co-producing safety and independent living. The study focuses on the experience of safety and independence, as these are expressed aims for the social alarm.

The research question examined: How do older people pursue, maintain, and negotiate independence and safety in everyday life by using social alarms?

## Methods

### Context, Settings, and Sample

The study was conducted within a homecare service in Norway in the period from August 2014 to December 2015, combining 1-week full-time participant observation in 2 municipalities and in-depth interviews (n=22) with actors involved in the use of social alarms. This provided a comprehensive picture of the respondents’ experiences with social alarms, promoting an in-depth understanding of this technology in use.

In Norway, all inhabitants are entitled to care services at home if required [2,47,48]. Two municipalities were strategically chosen for maximum diversity, representing both typical and diverse local communities in Norway as displayed in Table 2.

One municipal homecare manager in each municipality recruited participants for interviews. Older people, relatives, and care workers were selected to be interviewed according to the inclusion criteria described in Table 3.

A strategic participant selection was conducted aiming for maximum diversity. A description of included respondents is presented in Multimedia Appendix 1. The end users lived either in their own home or in care flats. The latter are small flats, often organized in proximity to a nursing home. The fieldwork involved participant observation in homecare services in both municipalities in the various cultural contexts. This entailed accompanying care personnel responsible for receiving alarm calls on their shifts while visiting end users.

The interviews were conducted with people possessing a social alarm (n=11), relatives of people possessing the alarm (n=4), care workers with different backgrounds and responsibilities (n=6), and key workers and managers (n=3). Some respondents had several roles. The 22 interviews were conducted in the respondents’ homes or at home care service facilities and lasted between 30 minutes and 2 hours.

In this study, the respondents told hundreds of stories, providing extensive material including the narratives presented later. All interviews were audio recorded and fully transcribed verbatim. NVIVO 11 (QSR International) software for data analysis was used as a tool when organizing, analyzing, and finding insights into the material.

### Interview Design

Critical incidence technique (CIT) methodology was applied, and the respondents were asked to describe incidents related to the social alarm. CIT is a practically oriented, commonly used explorative approach that facilitates insights into the complexities of an event and the interactions between actors involved [49,50]. This provided rich stories of critical incidents balanced with normal use descriptions. The method is attentive to the way practice is lived, generates rich material, and uncovers tacit understandings of an incident, including affective, cognitive, and behavioral elements [51,52]. Critical incidents can never be seen as isolated but rather as integrated into contexts. Narratives of lived experiences thereby emerged as presented in the results section. CIT is used within different research traditions, and this study is aligned with a phenomenological-interpretivist tradition as developed by Elizabeth Chell. Incidents are described as something emerging from the practice, embedded in the actor’s perspective [50,51,53].

### Ethics Approval and Consent to Participate

The Data Protection Official for Research in Norway granted approval for the project (project number 38605). All interview participants gave informed written consent. The municipal health care management gave written consent to collect data within the homecare services. All health care professionals received written and verbal information about the study and data collection and were informed that they could decline to be observed or interviewed. Prior to the observational study, the health care workers informed patients about the study and asked if researchers could observe the encounter.

**Table 2:** Characteristics of the municipalities included in the study.

Characteristics	Municipality 1	Municipality 2
Inhabitants	30,000	2600
Geography	Midsize city; inland	Rural district; northern coastal area
Responders	Homecare personnel	Call center that contacts homecare personnel when necessary

**Table 3:** Recruiting criteria for the interviews.

Respondents	Recruiting criteria
End users	Possessed a social alarm for more than 1 year Varying experiences with the alarm Both sexes, a variety of ages, living conditions, and dependency
Next of kin	Difference in relationships and living distance from the end user
Care workers	Experience with responding to the social alarm Varying professions and responsibility related to the alarm

### Analysis

Detailed observational field notes and transcriptions from the interviews were analyzed thematically using a stepwise deductive-inductive analysis described by Tjora [54], who was inspired by Strauss and Corbin [55,56]. The analytical focus was on participants’ perspectives and experiences in accordance with CIT [49].

The analysis started with an empirically close coding of the transcripts resulting in 68 codes focusing on participants’ perspectives. This inductive coding related to the actor’s experiences and co-production with the social alarm. This was further analyzed and grouped into 11 categories based on the actors’ voices [54]. Three themes of relevance to this paper formed the empirical-analytical basis: attaching to the alarm, interacting with others, and tinkering and bricolage. These themes embody how the different actors involved co-produce safety and independent living for the end users. The themes apply to all respondents in the study in various ways.

By going back and forth between the theory and the themes, 3 narratives emerged from this analysis, using Figure 2 as a sensitizing tool. Purposeful sampling provided a way of studying these rich cases in depth [57], as the narratives are suited for illustrating the themes from different perspectives and provide rich descriptions of different user styles. This illustrates how the social alarm opens up for different co-production practices and contributes to different experiences of safety and independent living when integrated into daily lives.

## Results

### Co-Producing Safety and Independent Living With the Social Alarm as a Life Saver

The 3 anonymized narratives presented reflect different unique lived experiences related to the social alarm and how safety and independent living are co-produced between the different actors and the social alarm. This illuminates different aspects of the empirical material corresponding to the 3 themes that emerged from the analysis.

The respondents recounted dramatic and possibly life-threatening events. Even so, this first narrative particularly stood out as “Anna,” her daughter “Turid,” and several of the care workers related it unsolicitedly.

Anna is a 96-year-old widow. She lives alone in her house in a depopulated rural area. She and one other person still live in the hamlet. She got the social alarm 16 years ago after suffering a stroke. She describes a strong attachment to the alarm and says that she feels safe, trusting that she can get help if needed. She has 5 adult children, all living far away. However, they speak daily on the phone, and someone always comes home during the holidays. Anna suffers from several chronic conditions, making her dependent and frail. However, she manages on her own and walks with a stick. This story took place in late wintertime when it was very cold and there was a lot of snow on the ground.

I was going outside with a bag of old newspapers. Then I fell on the ice. Broke my hip. I was lying there completely immobilized. I was wearing the alarm at the time; otherwise, I would not be here today. It was a quarter to ten in the morning when I fell, and they would not have started looking for me before six o’clock in the afternoon. However, I had the alarm, so I pressed the pendant and got help from the homecare nurse and ambulance in just a few minutes. I was conscious the whole time; the nurse came immediately because she was nearby. She went inside for a pillow and a blanket, which she laid under me until the ambulance came and took me to the hospital. The alarm saved my life that time, that’s for sure. And many other times as well, as things are. I would be dead by now for sure if I didn’t have the alarm.

Interacting with others was central for Anna’s use of the social alarm, and the collaboration with the care worker made her feel safe and attended to.

When Anna talked about her attachment to the alarm, she said, “The most precious thing I have is the social alarm.” She described how the alarm was crucial for her ability to feel safe and to be able to stay at home. She was not afraid for herself, she said. However, she did not want her children to worry.

Her daughter Turid said that she felt her mother was fairly safe, as long as she had the social alarm, as they had experienced that she got help when in need. When asked what the alarm means for her as next of kin she answered:

Very much. It really does. We do talk on the phone every day, but she has had a stroke you know. She is paralyzed on her right side. She cannot grip things with her right hand. And her right foot, she sways a bit, and overbalances easily. We are a bit insecure regarding her staying at home. She is clear-headed and wants to continue living at home. And as she says: I do have the alarm. Yes, but as I say, she has been lucky when falling, not falling on her left side. Then she would not be able to use the alarm. The last time she fell, I started to wonder how ethically right it is, we all feel that way.

Turid describes a fragile dependent mother as borderline in terms of whether she is able to live alone with the social alarm as a safety net—they are managing, but only just.

### Depending on the Social Alarm When Co-Producing Independence

Although many of the stories told in this study were dramatic, some are about undramatic but still decisive experiences. Some end users had never activated the social alarm due to emergencies; one of them is “Jon.”

Jon is a man in his early sixties with a progressive neurological disease that has partly restricted him to a wheelchair. His health is deteriorating, but he still manages to live in his house, with visits from the care worker for weekly medication. He appears emotional regarding his attachment to the social alarm and repeatedly praises it for making living at home possible.

...the possibility of having one of these [enthusiastically waves the pendant], especially when you live alone and are still going down to the basement for firewood. Then it’s crucial, the social alarm, because it actually works... Hurray for the alarm!

Jon has had a social alarm for some years now. He always has it in the pocket of a leather waistcoat with his mobile phone in the other pocket.

Jon describes himself as a very engaged, active man with a positive attitude. When Jon talks about his attachment to the alarm, he describes how it makes him feel safe and allows him to keep doing things without worrying about falling and not getting help. At the same time, he adds that he didn’t care for the alarm in the beginning. He regarded it as an indicator of his declining health and felt that by accepting the alarm, he was accepting deterioration. He needed time to get used to it before accepting it. This aligns with other studies describing how end users chose to use the alarm but did not care for it [36,58,59]. Jon had a brother with the same disease as himself. The two of them had different approaches to life with a disability, Jon said. While he really wants to manage on his own and stay active, his brother in contrast accepted his decline sooner and became more passive. One example is that Jon only uses his wheelchair when necessary, while his brother embraced the wheelchair and never got up again once he sat down.

Jon described how important it is for him to decide for himself and to be in a dialogue, interacting with the care service. He described meetings where care workers suggested that he moved to a care flat, but he wants to stay in his house. He also gets positive feedback from the care workers regarding his independence and humor and how he likes to make jokes.

Jon had never needed to activate the alarm due to an emergency, and he talked about how he sometimes felt insecure about whether it really worked and that he sometimes activates it just to check.

I succumbed to the temptation a few times and pressed the pendant. “Hello, this is me. If you are nearby, could you please come by? I have not been to the grocery store and need some warm food.” Then I started rattling out anything I could think I needed. They gradually started laughing. They know me, they’ve been here before and knew I’m hopeless that way. Black humor, you could say...

It was important for Jon to be valued as independent and with a sense of humor. The quote describes how he managed to maintain his humorous attitude toward the care personnel while at the same time co-producing safety by testing the social alarm. Moreover, the care personnel allowed him to do so. The alarm has automatic functional testing so manual testing could be regarded as unnecessary. However, this co-production made him feel safe and confirmed that he could trust the alarm. He was dependent on the alarm to be safe enough to live independently, even though he had never needed to activate it.

### Creative Co-Production With the Social Alarm

The last narrative is about “Peter” and “Marie.” Their daughter “Kari” told the story. Peter and Marie were an old married couple living in a care flat. People living there are usually dependent and frail, as were Peter and Marie, who moved from their house when their health was deteriorating. Peter tended to fall and he sometimes passed out due to a drop in blood pressure. Then his fingers got numb and he could not manage to press the pendant. Marie was the one wearing the alarm. She was physically vigorous, even though she had had a stroke some years back and suffered from dementia. Marie did not understand how she should call for help when her husband got ill, and Peter was too heavy for Marie to manage. Kari describes how her parents had a combined attachment to the social alarm.

When he experienced falls in blood pressure, he passed out for a while. Sometimes she pressed the pendant, sometimes not. She got confused when these things happened, but then he woke up and said, “Press the button!”

After they moved to the care flat, he fell several times. Sometimes he passed out, but not always. Then he told her to press the pendant. This could be early in the morning, or if he needed to get up to pee at night, ... and then she pressed the pendant. I don’t think she did it before he told her to. No, but then they got help, and I believe that if they hadn’t had the alarm, then he might have been lying there for 2-3 hours, maybe more. There are many examples like this.

Marie managed to press the alarm pendant when Peter instructed her. Together they managed to co-produce what neither of them managed alone by tinkering with the use of the alarm. Kari could not tell how this arrangement came about, but she thought it was done in collaboration between Peter, Marie, and the care workers.

Then Peter died. Marie still has the social alarm, but Kari does not believe her mother is capable of activating it when in need, as she has never activated it since her husband died. She is very dependent, and her dementia is progressing.

## Discussion

### Principal Findings

The key objectives of this paper were to explore and interpret how through interaction with the social alarm different actors co-produce safety and independent living for older people with the alarm. A further aim was to provide a framework for exploring co-production in care practices when technology is integrated.

The framework illustrated in Figure 2 provides theoretical tools to explore and interpret the co-production. This entails using the social alarm to pursue, maintain, and negotiate independence and safety for end users. Examples from the narratives presented in the paper illustrate different user styles and work and interaction with the social alarm in use as further presented.

### Attaching to the Alarm

Figure 2 presents scripting and domestication as tools for exploring the co-production of expectations and experiences with technology in use. It further illustrates how a technology in use can be scripted in dimensions related to the artifact, service, and values that come into play. When using Figure 2 as a sensitizing concept, the analysis revealed that the respondents in this study co-produce attachment to the alarm. This relates to expectations described in the scripting and experiences through the domestication process. The advocated scripting of the social alarm describes a technology that enables safe and independent living. Most respondents in this study agreed enthusiastically with this script. Jon’s story differentiates this attachment, as he did not care for the alarm at first, struggling with the less articulated script of it being suited for dependent and frail people. He interpreted it as a declaration of his deterioration. He gradually came to accept the alarm, now describing himself as dependent on the technology to stay independent. This illustrates the multiple scripting of the technology and how the relationship with the alarm might change over time through domestication. Exploring scripts and domestications provided insights into the co-production of expectations toward the social alarm and allowed us to articulate and explore the co-production of safety and independence in depth.

### Interacting With Others

Service innovation literature states that a service is always co-produced, as illustrated in Figure 2. So, what does this imply for the actors in their everyday practice?

The social alarm only works through co-production between the actors, including the technology as an actor. It is tenable and fragile and needs a collective, mutual persuasion in order to work and make sense. The person possessing the alarm needs to wear the pendant and activate the alarm to get help. The care worker has to answer the alarm calls and effect proper response. The active participation requirement largely means that responsibility for the safety of end users is delegated to the end users themselves, care workers, and the technology. This demonstrates how the social alarm is not “strong” in the sense of a powerful stand-alone technology. It also illustrates how the social alarm changes the dynamics between actors and technology and rearranges care practices.

Table 1 provided us with examples of different heuristics that arise in the interaction between human actors and technology. If we integrate examples from the narratives into Table 1, we find that these represent different heuristics, as described in Table 4.

Using Figure 2 enables us to focus on the script and domestication and how humans and technologies co-produce safety and independence in different ways, as illustrated in Table 4. Interpretative flexibility and contextual factors contribute to these different heuristics, and different practices emerge as we follow the suggestion of Pols [10] to study technologies in the context and network they are integrated into.

### Co-Production by Tinkering and Bricolage

The last column in Figure 2 provides us with a focus on how care is co-produced through the integration with the technology. Sometimes the technology does not quite fit the users’ needs or even their ability to handle the technology, and there is a need to work around this, creating bricolages. Bricolage comes into play in the co-production between end users and other actors as described in Table 4.

Bricolage appears when technology fails or does not meet the needs of the user, as when Jon activates the alarm to check whether it works. Bricolages also occur when the users do not meet the demands of the technology as illustrated by the example of Peter and Marie and their co-production of activating the alarm.

**Table 4:** Different practices illustrated by examples from the narrative.

Heuristics	Examples from the narratives of the co-production between technology, actors, and care service to meet the actors’ needs.	How actors involved co-produce care practices
Use in accordance with expectations	Anna and her family co-produce the interaction with the technology in accordance with the script, securing help when in need.	Co-producing safety and independence as expected by advocates of the social alarm and described in the scripts.
Taming the users	All narratives provide examples of how the end users are dependent on the technology but in very different ways. They are dependent on the social alarm to be able to live independently.	The users are able to co-produce the value of staying at home and feeling safe by interacting with the technology and other actors in the service.
Unleashing the users	Jon and the care workers co-produce a new service by allowing him to activate the alarm to check whether it works, even though testing is done automatically.	Using the alarm in an unpredictable way through bricolage. Co-producing safety and independence.
Taming the technology and unleashing practices	Peter and Marie are co-producing a way of taming the technology by co-producing ways of using the alarm. Both Marie and Peter’s collaboration and Jon’s workabouts are good examples of how the technology can unleash unexpected practices.	Co-production of independence and safety. By this, the time frame of use of the social alarm is extended through bricolage. If the municipality focuses on these unexpected co-production practices, they can use them for quality improvement and potential innovations in the service.
Nonuse	This was not relevant in this study as the research focused on the technology in use.	There is an important distinction between not activating the social alarm and not using the alarm, as we can see with Jon, who is dependent on the alarm although he has never activated it in an emergency.

Technology in care practices often has a time frame when it is possible for the users to use the technology, especially when the end users’ health is deteriorating as is often the case with elderly and chronically ill people [36]. The co-production between Marie and Peter made it possible to prolong the period of use. Neither of them was able to use the social alarm individually, but in collaboration, they were able to co-produce safety.

It is evident that vulnerability and even luck are involved in the success of the social alarm in use. Anna’s daughter describes how Anna is fortunate to have fallen on her right side. If she had fallen on her left side, she would not have been able to press the pendant due to paralysis. Luckily, the care worker was nearby when Anna broke her hip. If Peter had remained unconscious one day, Marie might not have been able to activate the alarm. It seems that sometimes there are narrow margins for the alarm to provide safety. This aligns with the argument of Procter and Greenhalgh [1] that although customization or bricolages might have advantages in the form of usability, they may also endanger patient safety.

Today these technologies are advocated as plug and play and one-size-fits-all user technology. This is an illusion. We should instead study the bricolage that emerges, as the stories illustrate, and make sure that quality is improved in a safe way for the end users. We would then be able to use more of the technology systematically in the long term and develop possible service innovations. One simple example is to copy Jon’s use by encouraging end users to press the pendant occasionally, giving them experience in activating the alarm and reassurance that it works.

To ensure quality in care practices, we need to ground the integration of the technology and other parts of the care practices in the end users’ lived experience [1].

Social alarms have been around for more than 30 years, and there are still ongoing processes of bricolage and co-production between actors to make them work. The technology is well established and thereby tends to be viewed as normalized and closed. However, when taking the actors and context in which the practice takes place into account, we find ongoing co-production processes where the users redefine and renegotiate the purpose and practical use of the alarm. According to Oudshoorn and Pinch [60], users will always find new ways of using familiar technologies. This bricolage can be seen as a kind of continuous service innovation, which seems to be a precondition for the integration of technology in everyday life.

### Conclusions

This paper focuses on the co-production of established technology in use. Using concepts from STS in the framework as presented in Figure 2 facilitated a comprehensive approach to studying technology integrated into care practices. The framework provided a tool to unpack and articulate the process of co-producing safety and independent living, which are the aims of social alarms. The study shows how practice involving an established simple technology may actually comprise complex practices that we need to thoroughly explore to understand. This would provide us with a more comprehensive picture of the interaction between them and the complexity at hand. The framework proved relevant for exploring technology innovations in care practices empirically.

Even with successful practices where the care and use of technology are grounded in the end users’ experience, bricolages are still seen. They should be regarded as possibilities for quality improvement in addition to tinkering with technology that does not work. Care work mainly consists of co-production between different actors in networks, with technologies as integrated components. Therefore, technology in use must be viewed as a collaborative activity.

This paper also reveals how the experience of the technology, as well as the possibility to use it, changes over time. Technology integrated into care practices can, therefore, not just be implemented and left alone to function; it must be adjusted and followed up as technology in use, constituting an everlasting process.

This study suggests a framework for exploring co-production when using technologies in care practices. However, this research is limited to social alarms, and several areas should be investigated further. This study focuses on examples where the technology mainly works and is successful. Empirical exploration of more complicated technologies going forward could promote better analytical clarity and contribute to the validation of the framework. Furthermore, studies of the changes in the different actors’ roles in the co-production of care practices would provide valuable insights.

## Appendix

Multimedia Appendix 1 Providing an overview of the included respondents.

## Abbreviations

CIT: critical incidence technique
STS: science and technology studies
