# Supplementary material for: Older People Negotiating Independence and Safety in Everyday Life Using Technology: Qualitative Study
Source: J Med Internet Res. 2018 Oct 19;20(10):e10054. doi: 10.2196/10054 (PMC6234346; doi:10.2196/10054)
Supplement: Multimedia Appendix 1 [file jmir_v20i10e10054_app1.pdf]

Multimedia appendix 1		
	Respondents in municipality 1	Respondents in municipality 2
Type of respondent		
The end user of the alarm		
	A widow, living alone in care flat in a home care centre<br><br>Widower living alone in his house<br><br>A widow living alone in her house<br><br>A widow living alone in her house<br><br>A single man living alone in his house	A widow living alone in care flat in a home care centre<br><br>A widow living alone in her house<br><br>Widower living alone in his house<br><br>A widow living alone in her house<br><br>A widow living alone in a home care studio flat
Next of kin		
	Close male friend<br><br>Daughter of a deceased user	Daughter living close<br>Daughter living far away
Care worker		
	Female nurse<br>Female assistant	Nighttime female nurse<br>Female assistant
The alarm manager		
	Female nurse assistant	Female nurse (same person as the home care manager)
Home care manager		
	Female nurse manager	Female nurse manager
